# CdTe_0.5_S_0.5_/ZnS Quantum Dots Embedded in a Molecularly Imprinted Polymer for the Selective Optosensing of Dopamine

**DOI:** 10.3390/nano9050693

**Published:** 2019-05-03

**Authors:** Kiana Khadem-Abbassi, Hervé Rinnert, Lavinia Balan, Zahra Doumandji, Olivier Joubert, Majid Masteri-Farahani, Raphaël Schneider

**Affiliations:** 1 Faculty of Chemistry, Kharazmi University, Tehran 15719-14911, Iran; nature.ph.111@gmail.com (K.K.-A.); mfarahani@khu.ac.ir (M.M.-F.); 2 Institut Jean Lamour, UMR CNRS 7198, Université de Lorraine, F-54000 Nancy, France; herve.rinnert@univ-lorraine.fr (H.R.); zahra-manel.doumandji@univ-lorraine.fr (Z.D.); olivier.joubert@univ-lorraine.fr (O.J.); 3 Institut de Science des Matériaux de Mulhouse, CNRS, UMR 7361, 15 rue Jean Starcky, 68093 Mulhouse, France; lavinia.balan@uha.fr; 4 Laboratoire Réactions et Génie des Procédés, Université de Lorraine, CNRS, LRGP, F-54000 Nancy, France

**Keywords:** quantum dots, molecularly imprinted polymer, dopamine, fluorescence, quenching

## Abstract

This work describes the preparation of molecularly imprinted polymer (MIP)-modified core/shell CdTe_0.5_S_0.5_/ZnS quantum dots (QDs). The QDs@MIP particles were used for the selective and sensitive detection of dopamine (DA). Acrylamide, which is able to form hydrogen bonds with DA, and ethylene glycol dimethylacrylate (EGDMA) as cross-linker were used for the preparation of the MIP. Highly cross-linked polymer particles with sizes up to 1 µm containing the dots were obtained after the polymerization. After the removal of the DA template, MIP-modified QDs (QDs@MIP) exhibit a high photoluminescence (PL) with an intensity similar to that of QDs embedded in the nonimprinted polymer (NIP). A linear PL decrease was observed upon addition of DA to QDs@MIP and the PL response was in the linear ranges from 2.63 µM to 26.30 µM with a limit of detection of 6.6 nM. The PL intensity of QDs@MIP was quenched selectively by DA. The QDs@MIP particles developed in this work are easily prepared and of low cost and are therefore of high interest for the sensitive and selective detection of DA in biological samples.

## 1. Introduction

2-(3,4-Dihydrophenyl)ethylamine, also called dopamine (DA), is a member of the catecholamine family. DA is a key neurotransmitter and regulates numerous physiological processes in cardiovascular, central nervous, and hormonal systems [1,2]. Abnormal concentrations in DA in biological fluids are indicative of diseases or neurological disorders including Parkinson’s and Huntington’s diseases or schizophrenia [3,4,5]. Hence, the development of sensitive and facile methods for DA detection are needed.

In recent years, a variety of methods have been implemented to detect DA including chemiluminescence [6,7], electrochemistry [8], colorimetry [9,10], liquid chromatography or capillary electrophoresis [11], and fluorescence spectroscopy [12,13,14,15,16]. Each of these methods exhibits advantages and drawbacks. For example, ascorbic acid (AA) and uric acid have a similar oxidation potential to DA and its selective detection by electrochemistry is problematic. Chromatographic methods are time-consuming and generally require complicated procedures. Moreover, the detection of DA at low concentrations (10 nM to 10 µM) is also a challenge. Fluorescence spectroscopy is a low cost and highly sensitive method for the detection of biomolecules and can thus be considered as an ideal method for DA sensing [12,13,14,15,16]. 

During the last two decades, molecularly imprinted polymers (MIPs) have been the subject of intensive research [17,18,19,20,21]. The molecular imprinting process is based on the synthesis of a polymer in the presence of a template, the target molecule, which allows the introduction of molecular recognition cavities in the polymer network. These cavities are specific in size and shape and exhibit high substrate recognition ability. Due to their high stability, ease of fabrication and low cost, MIPs have found applications in numerous fields like analytical chemistry, especially for the analysis of drugs, pesticides, peptides, biological or environmental samples, but also for the separation of molecules, for drug delivery and as sensors [17,18,19,20,21,22,23]. Recently, the potential of MIPs as synthetic receptors for cell recognition has also been demonstrated [24]. 

Quantum dots (QDs) are fluorescent nanocrystals with sizes generally less than 10 nm that exhibit unique optical and electronic properties such as size and composition tunable photoluminescence (PL) emission and high levels of photostability [25,26]. The high potential of QDs for applications like diagnostic, bio-imaging, and sensing has been demonstrated throughout the fifteen past years. Surprisingly, QDs embedded in MIPs have only scarcely been used for the fluorescent optosensing of DA. Molecularly imprinted silica nanospheres containing carbon dots were the first developed to detection DA with a limit of detection of 1.7 nM [27]. More recently, molecularly imprinted polyindole or poly(indolylboronic acid) functionalized graphene QDs were described for sensing DA with detection limits of 0.1 and 2.5 nM, respectively [28,29].

The present study reports an efficient and facile strategy for DA detection by using a novel composite of core/shell CdTe_0.5_S_0.5_/ZnS QDs [30] enwrapped in a MIP engineered from acrylamide and ethylene glycol dimethylacrylate (EGDMA), used as cross linker. The polymerization was initiated using potassium persulfate K_2_S_2_O_8_. The DA template was removed by disrupting the hydrogen bonds between the amine and hydroxyl groups of DA and the carbonyl functions of acrylamide, leaving 3D-cavities that were complementary to the molecular shape of DA. We demonstrate that the prepared QDs@MIP composite exhibits a high ability to selectively detect DA over other common molecules in the cell, including amino acids, peptides, proteins, vitamins, ions, and other neurotransmitters. The mechanism of the PL quenching was also investigated. Finally, it should also be mentioned that the acrylamide/acrylate MIP surrounding CdTe_0.5_S_0.5_/ZnS QDs is prepared from readily available, air-stable, and low-cost monomers, which is a major advantage compared to QDs@MIP particles developed to date for the detection of DA.

## 2. Materials and Methods 

### 2.1. Chemicals

Cadmium chloride hemi(pentahydrate) (CdCl_2_·2.5H_2_O, >99%), zinc acetate dihydrate (Zn(OAc)_2_·2H_2_O, >99%), tellurium (Te, >99.8%), sodium borohydride (NaBH_4_, >96%), 3-mercaptopropionic acid (MPA, 99%), acrylamide (>98%), ethylene glycol dimethylacrylate (EGDMA, 98%), potassium persulfate (K_2_S_2_O_8_, >99%), dopamine hydrochloride (DA·HCl, 99%), adenosine 5′-diphosphate (ADP, >95%), l-arginine (Arg, >98%), l-histidine monohydrochloride monohydrate (His, >98%), l-lysine (Lys, >98%), l-serine (Ser, >99%), l-cysteine (Cys, 97%), l-tyrosine (Tyr, >98%), glycine (Gly, >98.5%), homovanillic acid (HVA, fluorimetric reagent), l-glutathione (GSH, >98%), bovine serum albumin (BSA, >96%), (d)-(+)-glucose (>99.5%), ascorbic acid (AA, >99%), l-aspartic acid (Asp, >98%), KCl (>99%), NaCl (>99%), CaCl_2_ (>96%), and MgCl_2_·6H_2_O (>99%) were purchased from Sigma-Aldrich (Saint-Quentin Fallavier, France) and were used without purification. All solutions were prepared with deionized water (18.2 MΩ cm). Phosphate-buffered saline (PBS) solution was prepared using [Na_2_HPO_4_·2H_2_O] = 0.2 M, [NaH_2_PO_4_·H_2_O] = 0.2 M and the final pH was adjusted to 7.4.

### 2.2. Synthesis of Core/Shell CdTe_0.5_S_0.5_/ZnS QDs

The synthesis of core/shell CdTe_0.5_S_0.5_/ZnS QDs was conducted accordingly to the procedure we recently developed [30]. Briefly, CdCl_2_·2.5H_2_O (114 mg, 0.5 mmol) and Zn(OAc)_2_·2H_2_O (109.7 mg, 0.5 mmol) were dissolved in 50 mL of ultrapure water and MPA (105 μL, 2.4 mmol) was added. The pH of this solution was adjusted to 11.2 by dropwise addition of a 1 M NaOH solution. The solution was then placed in a 100 mL three-necked flask equipped with a thermometer and a condenser and was deaerated by bubbling argon for 1 h. Next, 6.25 mL of a freshly prepared aqueous solution of NaHTe (0.04 M) were quickly injected and the reaction mixture was heated to 100 °C under argon flow. After cooling to room temperature, the crude solution of QDs was used without purification for the next step. 

### 2.3. Synthesis of CdTe_0.5_S_0.5_/ZnS@MIP and CdTe_0.5_S_0.5_/ZnS@NIPs Sensors

A three-neck flask equipped with a condenser was filled with 30 mL of CdTe_0.5_S_0.5_/ZnS QDs aqueous solution (with a pH of 11 and a concentration of ca. 3 mg/mL), 310 mg of acrylamide, and 34 mg of EGDMA and the mixture was deaerated for 30 min. The temperature was raised to 70 °C and 35 mg of K_2_S_2_O_8_ were added to initiate the polymerization. After 1 h, 30 mg of DA hydrochloride was added and the mixture was further stirred for 2 h at 70 °C. Noteworthy is that at pH 11, DA·HCl is converted into DA and thus DA should be considered at the real template introduced in the MIP. After completion of the reaction, the system was cooled down to room temperature. The QDs@MIP particles were recovered by centrifugation (4000 rpm for 15 min) and washed with deionized water in order to remove CdTe_0.5_S_0.5_/ZnS QDs unbound to the MIP and unreacted monomers. The obtained QDs@MIP composite was first washed under sonication using an acetonitrile/methanol/water mixture (volume ratio of 1:1:3) and then with water until no DA could be detected from the washing solvents by UV-visible spectroscopy (Thermo Fisher, Illkirch, France). QDs associated to the nonimprinted polymer (NIP) were prepared using a similar synthetic protocol but without adding the DA template. QDs@MIP and QDs@NIP particles were stored as wet powders and at 4 °C for further use. The fluorescence intensity and the PL emission peak remain unchanged for at least two months storage at 4 °C.

### 2.4. Detection of DA in Aqueous Solution

Stock solutions of QDs@MIP and QDs@NIP particles with a 1 mg/mL concentration were prepared by dispersion of the particles in PBS (100 mM, pH 7.4). In preliminary experiments not described herein, the influence of QDs@MIP concentration on the sensitivity and selectivity for DA detection was investigated. Results obtained show that the concentration of QDs@MIP particles could be decreased up to 4.3 ng/mL without alteration of the detection toward the template molecule. 

For DA detection, QDs@MIP and QDs@NIP were dispersed under sonication in deaerated PBS at a concentration of 4.3 nanogram/mL. Neutralized DA (stored under inert atmosphere) was then added to 2 mL of the QDs@MIP or QDs@NIP solution in PBS and the mixture sonicated for 40 min. Then, the mixture was transferred into a quartz cuve and the fluorescence was measured using an excitation wavelength of 375 nm.

### 2.5. Selectivity of DA Detection

20 µL of a 2.63 mM solution of DA, ADP, Arg, His, Lys, Ser, Cys, Tyr, Gly, HVA, GSH, BSA, AA, Asp, KCl, NaCl, CaCl_2_ or MgCl_2_ in PBS (100 mM, pH 7.4) were added to 2 mL of QDs@MIP (concentration of 4.3 nanogram/mL) in PBS. The mixture was magnetically stirred for 40 min, transferred into a quartz cuve and the fluorescence was measured using an excitation wavelength of 375 nm.

### 2.6. Biocompatibility

*Cell culture.* Human THP-1 monocytic cell line was obtained from American Type Culture Collection (ATCC, TIB-202TM, Manassas, VA, USA). Cells were grown at 37 °C under 5% CO_2_ atmosphere in RPMI 1640 medium supplemented with 10% of heat-inactivated fetal bovine serum, 100 U/mL of penicillin, 100 µg/mL of streptomycin and 0.25 µg/mL of amphotericin. They were split every three days. 

*Cell viability.* Cell viability assay was analysed using the WST-1 assay (Roche, 11644807001, Meylan, France), according to manufacturer’s protocol [31]. THP-1 cells were seeded at 5 × 10^4^ cells/mL in 96-well plates and exposed to different concentrations of MIP-capped CdTe_0.5_S_0.5_/ZnS QDs. After 24 h of exposure, WST-1 reagent was added in each well. Cells were incubated at 37 °C for 2 h. The absorbance of the solution was determined at 480 nm on microreader (BioRad-iMARK, Marnes la Coquette, France) to calculate the IC_50_ for each QD. Each experiment is carried out on three independent biological replicates.

### 2.7. Characterization 

Transmission electron microscopy (TEM) images were taken by placing a drop of the particles dispersed in water onto a carbon film-supported copper grid. Samples were studied using a CM200 instrument operating at 200 kV (Philips, Suresnes, France). The X-ray powder diffraction (XRD) diagrams were measured using Panalytical X’Pert Pro MPD diffractometer (Malvern, Orsay, France). The powder samples were placed on a silicon zero-background sample holder and the XRD patterns were recorded at room temperature using Cu K_α_ radiation (λ = 0.15418 nm). 

All the optical measurements were performed at room temperature (20 ± 1 °C) under ambient conditions. FT-IR spectra were recorded on a ALPHA spectrometer (Bruker, Palaiseau, France). Absorption spectra were recorded on a Evolution 220 UV-visible spectrophotometer (Thermo Fisher, Illkirch, France). Photoluminescence emission spectra were measured on a Fluoromax-4 spectrofluorimeter (HORIBA Jobin Yvon, Longjumeau, France) PL spectra were spectrally corrected and PL QYs were determined relative to Rhodamine 6 G in ethanol (PL QY = 94%).

For the time resolved photoluminescence (TR-PL) experiments, the QDs were irradiated by the 355 nm line of a frequency-tripled YAG (yttrium aluminium garnet):Nd laser. The laser pulse frequency, energy and duration were typically equal to 10 Hz, 50 µJ and 10 ns, respectively. The PL signal was analyzed by a monochromator equipped with a 600 grooves/mm grating and by a photomultiplier tube cooled at 190 K. The rise time of the detector is equal to around 3 ns. 

## 3. Results

### 3.1. QDs@MIP Synthesis and Characterization

**Scheme 1:**
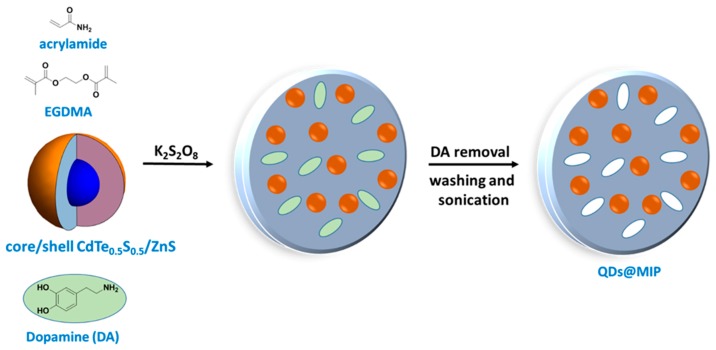
Schematic illustration of the preparation of molecularly imprinted polymer-modified core/shell CdTe_0.5_S_0.5_/ZnS quantum dots (QDs@MIP) particles.

QDs@MIP particles were prepared by K_2_S_2_O_8_-mediated copolymerization of acrylamide and EGDMA in the presence of the QDs and DA (Scheme 1). The addition of DA before initiation of the polymerization caused an irreversible quenching of QDs fluorescence, likely due to the strong bounding of DA at the surface of the dots. This irreversible quenching was not observed when DA was added 1 h after the start of the polymerization. After an additional 2 h of reaction followed by cooling, the DA template was removed by washing under sonication of the QDs@MIP particles with an acetonitrile/methanol/water mixture and then with water. The PL of QDs was restored after the template removal (*vide infra*).

**Figure 1:**
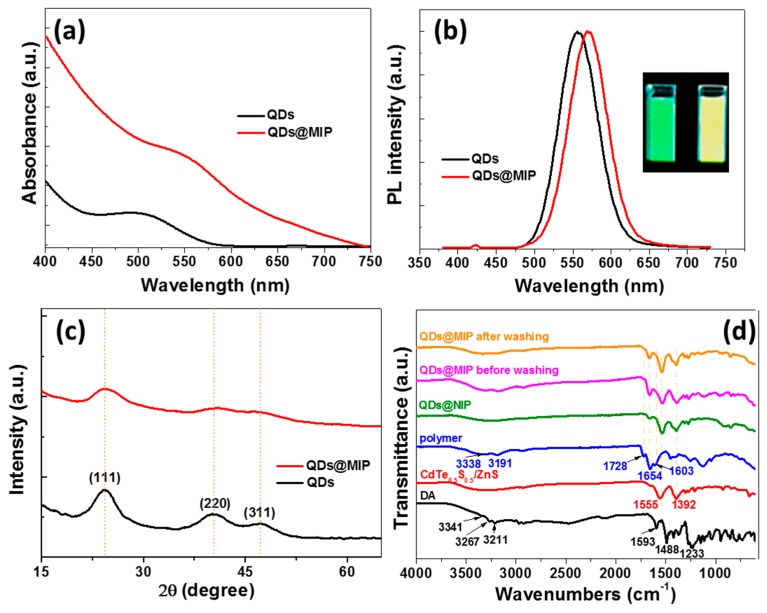
(**a**) UV-visible, (**b**) photoluminescence (PL) emission spectra (the inset of Figure 1b is a digital photograph taken under UV-light irradiation of QDs and QDs@MIP) and (**c**) XRD patterns of CdTe_0.5_S_0.5_/ZnS QDs and of QDs@MIP. (**d**) FT-IR spectra of DA, CdTe_0.5_S_0.5_/ZnS QDs, polymer, QDs@NIP and QDs@MIP before and after template removal.

The UV-visible absorption maximum of MPA-capped CdTe_0.5_S_0.5_/ZnS QDs is located at 500 nm and the dots emit at 557 nm after excitation at 375 nm (Figure 1a,b). Their PL quantum yield in water is 37%. After the growth of the copolymer at the periphery of the QDs, both the absorption and the PL emission maximum shift to longer wavelengths (λ_abs_ = 546 nm and λ_em_ = 571 nm). This red-shift of QDs absorption and PL emission likely originates from their further growth during the 3 h of polymerization at 70 °C. The inset of Figure 1b shows a digital photograph taken under UV light illumination of the native green-emitting QDs and the yellow-emitting MIP-capped QDs after template removal. The full-width at half-maximum (ca. 60 nm) of the PL emission peak is not altered by this red-shift of the PL. A slight decrease of the PL quantum yield (27%) was observed after the polymer growth around the QDs.

The XRD patterns of starting QDs and of MIP-capped QDs are plotted in Figure 1c. The peaks at 24.08, 40.48, and 47.28° can be ascribed to (111), (220), and (311) crystals planes of the cubic zinc blende structure [30]. After surface imprinting, the width at half-maximum and the intensities of these signals decrease suggesting that an amorphous layer of MIP surrounds CdTe_0.5_S_0.5_/ZnS QDs. 

The embedding of QDs into the MIP was further confirmed by FT-IR spectroscopy (Figure 1d). For DA, the signals at 3267 and 3211 cm^−1^ correspond to the N–H stretching vibrations while the peak at 3341 cm^−1^ likely originates from the aromatic O–H stretching vibration. The peaks at 1593 and 1488 cm^−1^ can be assigned to the C=C stretching. Two strong peaks at 1555 and 1392 cm^−1^ corresponding to the asymmetric and symmetric stretch of the carboxylate function, respectively, can be observed for MPA-capped CdTe_0.5_S_0.5_/ZnS QDs. The copolymer obtained from acrylamide and EGDMA exhibits the typical peaks of N–H stretching vibrations (3338 and 3191 cm^−1^), amide C=O stretching (1654 cm^−1^) and of amide ending (1603 cm^−1^). The C=O stretching of the ester function of EGDMA appears at 1728 cm^−1^. The signals of the MPA ligand and of the copolymer can be observed for the composite prepared in the absence and in the presence of DA, indicating that DA doesn’t perturb the copolymerization. The signals of DA can hardly be seen in the QDs@MIP material before washing likely due to the low amount of DA incorporated in the poly(acrylamide/EGDMA) network. 

**Figure 2:**
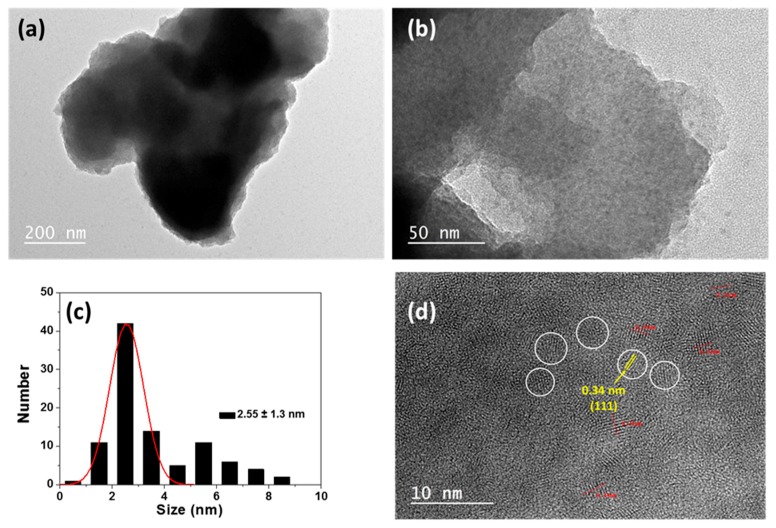
(**a**,**b**) TEM images of QDs@MIP particles, (**c**) the QDs size distribution and (**d**) HR-TEM images of QDs embedded in the MIP.

The successful preparation of QDs@MIP was further confirmed by TEM and high resolution-TEM (HR-TEM). MPA-capped CdTe_0.5_S_0.5_/ZnS QDs are spherical in shape and almost uniform in size with an average diameter of 2.6 ± 0.5 nm [30]. The copolymer growth embeds the QDs into larger particles with sizes up to 1 µm (Figure 2a) containing the dots (Figure 2a,b). After capping with the MIP, the size distribution of the dots is broader (2.6 ± 1.3 nm), which is in good agreement with the PL redshift observed (Figure 1b) but their crystallinity is not altered. Well resolved lattice fringes (interplanar spacing *d* = 0.34 nm) can be observed in the HR-TEM image, further confirming the cubic structure of the nanocrystals. The EDX spectrum of QDs@MIP particles is given in Figure S1. The strong atomic peaks of Cd, Zn, Te, and S and of the C element of the polymer further confirm the successful preparation of QDs@MIP particles.

### 3.2. Sensitivity of CdTe_0.5_S_0.5_/ZnS @MIP Particles for DA Detection and Mechanism

After the removal of the DA template, the fluorescence of QDs@MIP was restored and was found to be of similar intensity than that of NIP-coated QDs. To demonstrate the ability of QDs@MIP to detect DA, the PL response of the QDs@MIP composite was evaluated after adding DA at various concentrations. In preliminary experiments, the PL intensity of QDs@MIP was measured each 5 min after the addition of DA and the maximum quenching was observed after 40 min mixing indicating that molecular imprinted cavities in the MIP, complement to DA in size, shape, and spatial arrangement, have a high sensitivity toward DA due to specific interactions. In further experiments, a contact time of 40 min between the QDs@MIP and DA will be used before the PL measurement.

**Figure 3:**
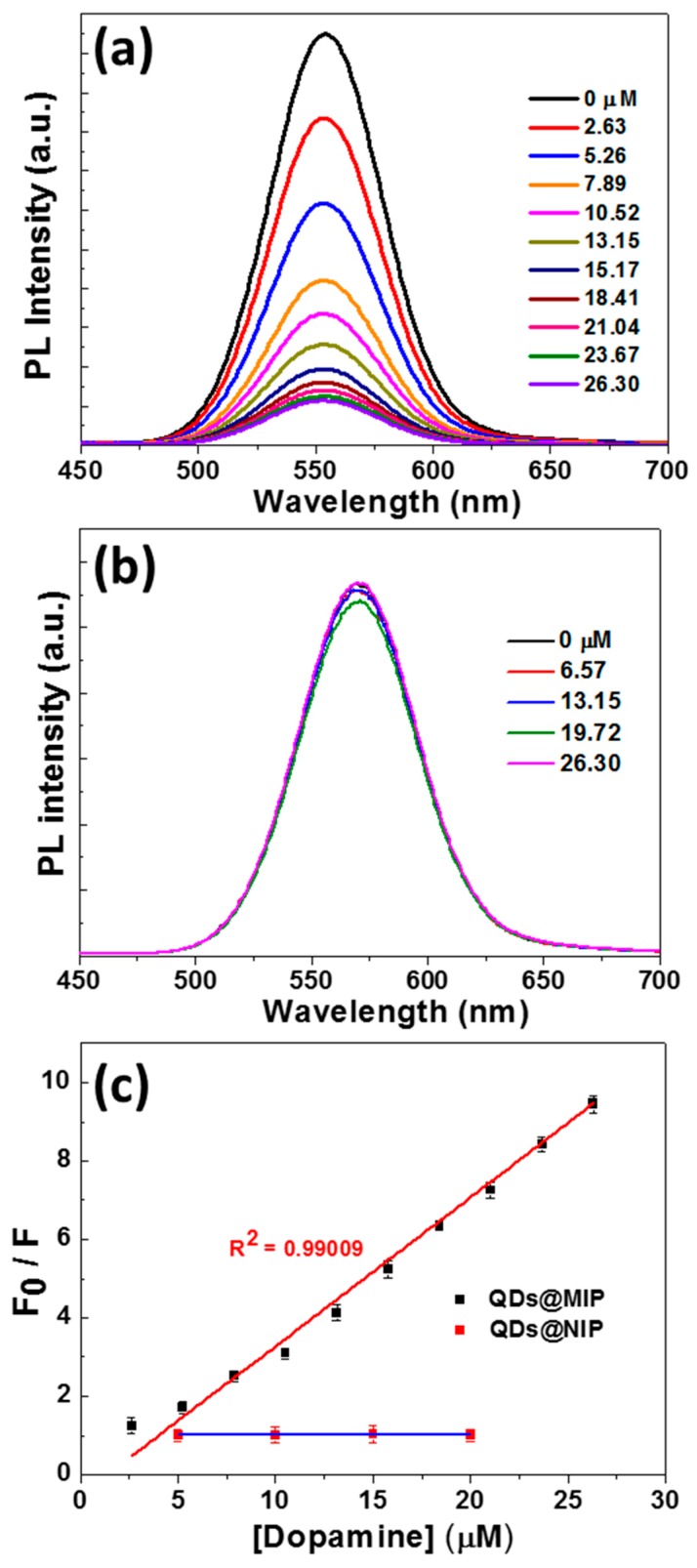
Fluorescence response of (**a**) QDs@MIP and (**b**) QDs@NIP particles with increasing concentrations of dopamine (DA). (**c**) Stern–Volmer description of the data obtained with QDs@MIP particles showing a linear fit throughout the DA concentration range. The calibration curve obtained with QDs@NIP particles is also given for comparison.

As can be seen in Figure 3a, the PL intensity of QDs@MIP decreased monotonically when increasing the concentration of DA from 2.63 to 26.30 µM while that of QDs@NIP remained almost stable (Figure 3b) because no selective recognition sites are present in QDs@NIP particles. These results confirm that the appropriately designed MIP markedly enhances the PL quenching efficiency of the dots in the presence of DA. The PL quenching of QDs@MIP follows the Stern–Volmer (SV) Equation (1):

*F_0_*/*F* = 1 + *K*sv [DA] (1)

where *F_0_* and *F* are the fluorescence intensities in the absence and presence of DA, respectively, *K*sv is the Stern–Volmer constant representing the affinity between the fluorophore and the quencher and [DA] is the concentration in DA. The *K*_SV_ value determined for QDs@MIP particles is 0.28 L·mol^−1^. A linear response was observed for concentrations in DA varying between 2.63 to 26.30 µM with a correlation coefficient R^2^ of 0.99. The relative standard deviations of three independent experiments vary between 0.3% and 3.5%, indicating a stable and reproducible response for the QDs@MIP sensor.

The imprinting factor (IF), defined as the ratio of [*K*_SV_ for QDs@MIP/K_SV_ for QDs@NIP], was calculated to be 104.86, a value confirming the high sensitivity of QDs@MIP for DA. The limit of detection (LOD) of the method was determined using Equation (2):

LOD = 3*σ*/*S* (2)

where *σ* is the standard deviation of the lowest tested concentration and *S* is the slope of the linear calibration plot and was found to be 6.6 ± 2 nM. The concentration of DA in most organisms is low (40 to 26 nM or even lower) [13], thus QDs@MIP particles developed herein may be of high interest for the detection of DA in living systems. The LOD value is slightly higher than those measured for carbon or graphene QDs@MIP developed for the DA detection (LOD varying between 0.1 and 2.5 nM) [27,28,29]. However, it should be noted that CdTe_0.5_S_0.5_/ZnS QDs@MIP can be used at a very low concentration (4.3 ng/mL) while carbon or graphene QDs@MIP were used at concentrations varying between 50 and 0.5 µg/mL.

**Figure 4:**
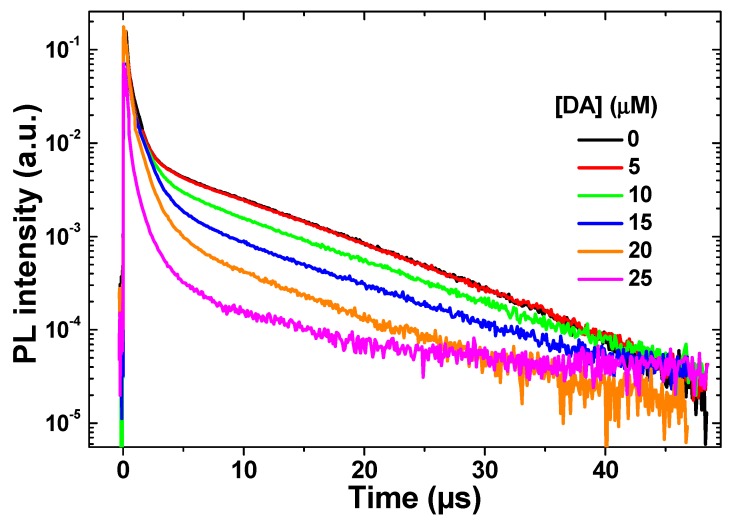
Time-resolved luminescence of QDs@MIP vs DA concentration measured at 550 nm.

To better understand the quenching mechanism of the PL emission, the time-resolved PL decay curves of QDs@MIP dispersed in PBS were recorded before and after the addition of DA (Figure 4). The PL decay curves measured at the maximum PL peak (λ_ex_ = 355 nm) can be fitted using a bi-exponential function *I*(*t*) = A_1_ exp (−*t*/τ_1_) and A_2_ exp (−*t*/τ_2_) characterized by the time constants τ_1_ and τ_2_ given in Table 1 (A_1_ and A_2_ the normalized amplitudes of the components). For QDs like CdTe, PL lifetimes are generally in the order of a few tens of nanoseconds [32,33,34]. In the absence of DA, QDs@MIP exhibit τ_1_ and τ_2_ values of 0.65 and 9.36 µs, respectively. These high lifetimes compared to CdTe QDs originate from the thickness of the ZnS shell and from the polymer network surrounding the CdTe_0.5_S_0.5_ cores, as previously observed for SiO_2_-coated CdTe nanocrystals [35]. The long lifetimes clearly indicate a delocalization of electrons after their excitation and the involvement of surface-states in their recombination [36]. Upon increasing the concentration of DA from 5 to 25 µM, the fast decay τ_1_ was not significantly affected but the slow decay τ_2_ decreased from 9.36 to 3.10 µs (Table 1). The marked decrease of τ_2_ demonstrates the high sensitivity of QDs@MIP toward DA.

The increase of the τ_0_/τ ratio (where τ_0_ and τ are the PL lifetimes in the absence and in the presence of DA, respectively) is typical of a dynamic quenching mechanism. A constant PL lifetime should be observed if a static quenching would be responsible of the PL decrease [37]. Since DA is easily oxidized into DA quinone in oxygen-containing solutions, it is likely that the quenching observed originates from an energy transfer from QDs to DA quinone associated to the MIP via hydrogen bonds.

### 3.3. Selectivity of QDs@MIPs for DA Detection and Toxicity

**Figure 5:**
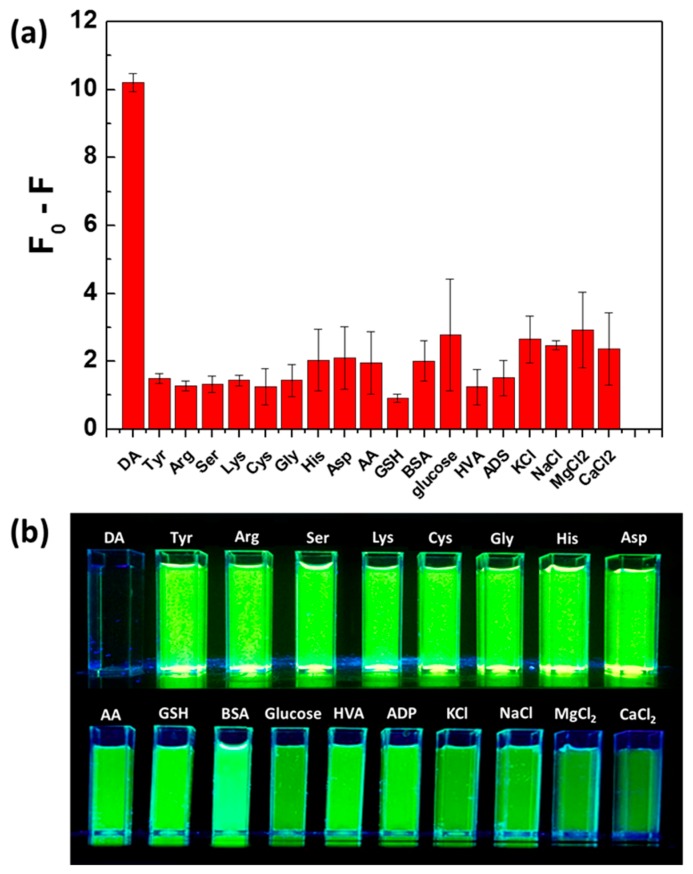
(**a**) QDs@MIP response toward DA and various potential interfering substances. QDs@MIP particles were used at a concentration of 4.3 ng/mL while DA and other substances were used at a 26.3 μM concentration. F_0_ and F are the PL intensities of QDs@MIP particles before and after adding DA or the potential interfering substances, respectively. (**b**) Digital photograph taken under UV light irradiation of PBS dispersions of QDs@MIPs after adding DA or the potential interfering substances (contact time of 40 min).

Since the QDs@MIPs particles are intended to be used for detecting DA, we also evaluated the possible PL quenching by other molecules present in the cells. As shown in Figure 5a, a strong PL quenching was observed when adding DA, whereas other molecules, such as amino acids (including Arg, His, Lys, Ser, Cys, Asp, and Tyr), peptide (GSH), protein (BSA), vitamins (AA), neurotransmitters (ADP, HVA, and Gly), glucose, and ions (including K^+^, Na^+^, Mg^2+^, and Ca^2+^), caused much weaker PL decrease. The digital photograph of aqueous dispersions of QDs@MIP after adding DA or the potential interfering substances confirms these results and further demonstrates that QDs@MIP particles can be used to selectively detect DA in aqueous solution (Figure 5b).

**Figure 6:**
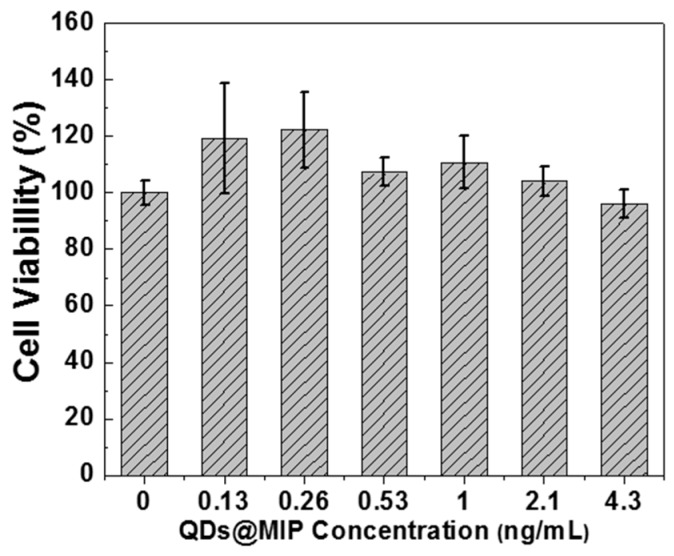
WST-1 assay on THP-1 cell line when varying the concentration of QDs@MIP particles from 0.13 to 4.3 ng/mL. Values represent means ± S.E (*n* = 3).

Finally, because the QDs@MIP particles contain cadmium, it is of high importance to study their biocompatibility in order to demonstrate that these materials can be used for various biological applications without causing cellular alterations. The THP-1 cell line was chosen in this study because circulating monocytes are considered as the first barrier of the organism against nanoparticles or bacteria [38,39]. Moreover, THP-1 cells are considered as an immune in vitro cell model and are validated for nanotoxicological studies [40]. The WST-1 assay, which is correlated to the metabolic activity of cells (mitochondrial succinate deshydrogenase function), demonstrates that QDs@MIP particles exhibit no cytotoxicity toward THP-1 cells. Figure 6 shows that cells remained alive and active even if the QDs@MIP concentration is higher than 4.3 ng/mL. The viability values above 100% originate from the hormesis phenomenon in which a xenobiotic induces a small rise of cell activity [40]. It is also noteworthy that the 4.3 ng/mL concentration is equal to that of QDs@MIP particles used for DA detection, further highlighting the potential of these particles for biological applications.

## 4. Conclusions

In summary, QDs@MIP microparticles were efficiently prepared from MPA-capped CdTe_0.5_S_0.5_/ZnS QDs, acrylamide, and EGDMA and used as recognition material for the detection of DA. The synthesis of QDs@MIP is facile and of low cost and these particles are highly sensitive to DA. A strong PL quenching likely originating from energy transfer from photo-excited QDs to DA-quinone was observed upon the addition of DA. The PL quenching was demonstrated to be linearly proportional to the DA concentration in the range from 2.63 to 26.30 µM with a limit of detection of 6.6 nM. Moreover, the particles can be used at a very low concentration (4.3 ng/mL) for the sensing of DA and no cytotoxicity was observed. QDs@MIP particles were also demonstrated to be of high selectivity and a much weaker PL quenching was observed in the presence of potential interfering substances like amino acids, ascorbic acid, or homovanillic acid. Due to their high sensitivity and selectivity, the QDs@MIP particles developed in this work may also be of interest for the detection of other biomolecules or for environmental monitoring.

## Figures and Tables

**Table 1:** Time constants τ_1_ and τ_2_ of QDs@MIP particles vs DA concentration.

[DA] (µM)	τ_1_ (µs)	τ_2_ (µs)
0	0.65	9.4
5	0.64	8.5
10	0.80	8.0
15	0.97	8.5
20	0.82	6.8
25	0.50	3.1

## Supplementary Materials

The following are available online at https://www.mdpi.com/2079-4991/9/5/693/s1, Figure S1: EDX spectrum of CdTe_0.5_S_0.5_/ZnS@MIP particles.
