# Supplementary material for: CdTe0.5S0.5/ZnS Quantum Dots Embedded in a Molecularly Imprinted Polymer for the Selective Optosensing of Dopamine
Source: Nanomaterials (Basel). 2019 May 3;9(5):693. doi: 10.3390/nano9050693 (PMC6566530; doi:10.3390/nano9050693)
Supplement: Supplementary file 1 [file nanomaterials-09-00693-s001.pdf]

SUPPORTING INFORMATION

$CdTe_{0.5}S_{0.5}/ZnS$ Quantum Dots Embedded in a Molecularly Imprinted Polymer for the Selective Optosensing of Dopamine

Kiana Khadem-Abbassi [1], Hervé Rinnert [2], Lavinia Balan [3], Zahra Doumandji [2], Olivier Joubert [2], Majid Masteri-Farahani [1] and Raphaël Schneider [4,*]

1 Faculty of Chemistry, Kharazmi University, 15719-14911 Tehran, Iran; nature.ph.111@gmail.com (K.K.-A), mfarahani@khu.ac.ir (M.M.-F.)

2 Institut Jean Lamour, UMR CNRS 7198, Université de Lorraine, F-54000 Nancy, France; herve.rinnert@univ-lorraine.fr (H.R.), zahra-manel.doumandji@univ-lorraine.fr (Z.D.), olivier.joubert@univ-lorraine.fr (O.J.)

3 Institut de Science des Matériaux de Mulhouse, CNRS, UMR 7361, 15 rue Jean Starcky, 68093 Mulhouse, France; lavinia.balan@uha.fr

4 Laboratoire Réactions et Génie des Procédés, Université de Lorraine, CNRS, LRGP, F-54000 Nancy, France;

* Correspondence: raphael.schneider@univ-lorraine.fr; Tel.: +33-372743790

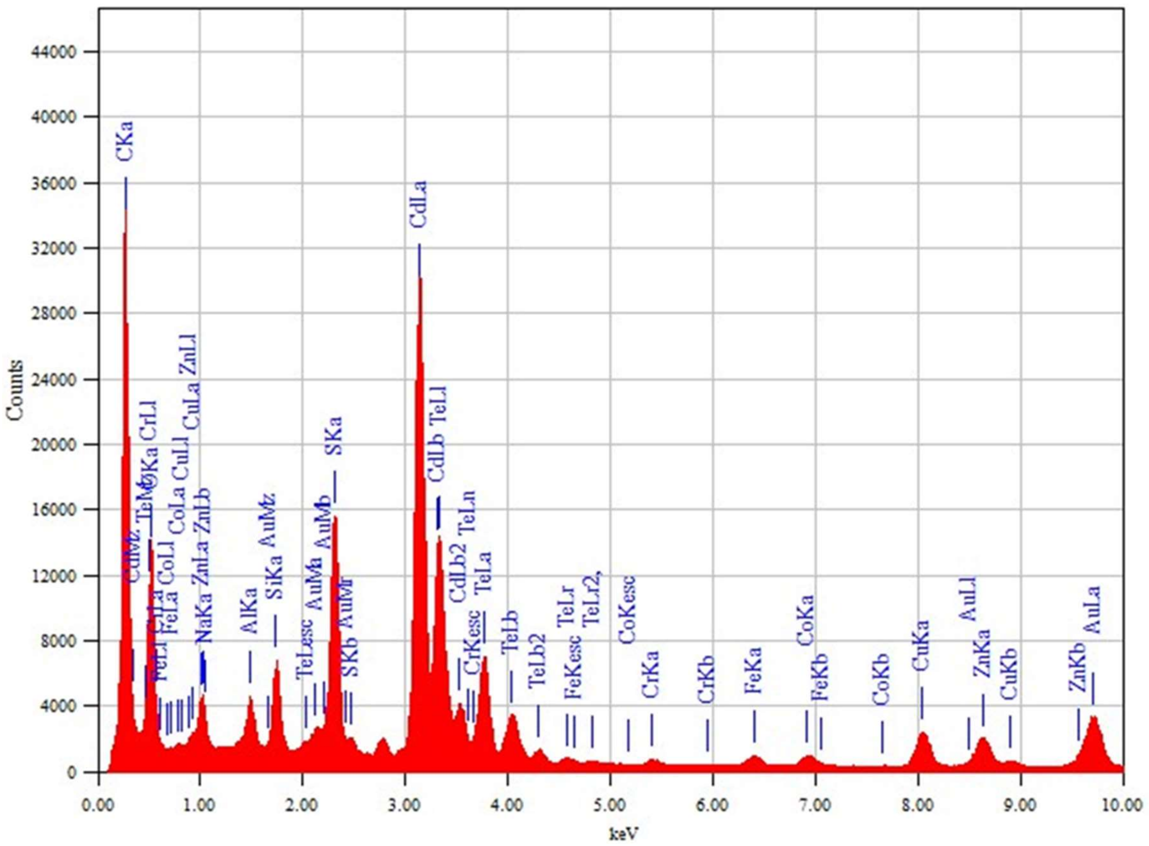

Figure S1. EDX spectrum of $CdTe_{0.5}S_{0.5}$/ZnS@MIP particles.
